# Protective effect of vaccinating infants with a 5 µg recombinant yeast-derived hepatitis B vaccine and the need for a booster dose in China

**DOI:** 10.1038/s41598-020-75338-5

**Published:** 2020-10-23

**Authors:** Ning Miao, Hui Zheng, Xiaojin Sun, Guomin Zhang, Fuzhen Wang

**Affiliations:** Chinese Center for Disease Control and Prevention, Nanwei Road, Xicheng District, Beijing, China

**Keywords:** Immunology, Diseases, Health care, Medical research

## Abstract

In 2002, China integrated hepatitis B vaccine (HepB) into its Expanded Program on Immunization (EPI) using HepB vaccine containing 5 µg of antigen. Although not recommended nationally, there was a common clinical practice in China of screening children for anti-HBs antibody level and giving a booster dose to HBV surface antigen (HBsAg)-negative children with non-protective anti-HBs antibody levels. We report an evaluation of the protective effectiveness of the 5 µg HepB vaccine and the serological response to the booster dose. We used data from a 2014 hepatitis B serological survey to determine HBsAg positivity and anti-HBs antibody levels among children who received and did not receive a booster dose. We determined HepB coverage from the Children Immunization Information Management System (CIIMS). We obtained and analyzed reports of acute Hepatitis B (AHB) during 2008–2014 obtained from the National Notifiable Disease Reporting System (NNDRS). The HBsAg-positive rate among children who had not received a booster dose was 0.41%, and did not increase with age (i.e., time since infant immunization). The anti-HBs positivity rate among the 6% of children who received a booster dose (88.41%) was higher than among those who had not received a booster (60.85%); anti-HBs antibody levels declined with age regardless of booster dose status. There was no statistically significant difference in HBsAg positivity between children who received a booster dose and those who did not. The AHB incidence among children born between 2002 and 2007 did not increase with age. Use of routine 5 µg HepB vaccine was not associated with an increase in AHB or of HBsAg positivity by time since vaccination, providing supportive evidence that individuals vaccinated with the 5 µg HepB vaccine do not need a booster dose. Although a booster dose was associated with increases in anti-HBs antibody levels, our study provided no evidence to support the need for this clinical practice. We should continue to strengthen serological monitoring of children, especially for those born to HBsAg positive mothers.

## Introduction

An estimated 257 million people were living with chronic hepatitis B virus (HBV) infection around the world in 2015^1^. HBV has been highly endemic in China, as historically, vertical HBV transmission built a reservoir of approximately 86 million chronically infected persons, accounting for 30% of the global burden of chronic HBV^2^. Serosurveys conducted in 1979 and 1992 in China showed a 10% prevalence of HBV surface antigen (HBsAg) positivity^3^. Universal administration of a timely birth dose of hepatitis B vaccine (HepB) followed by two more doses during infancy has been the key strategy to prevent HBV transmission in China. Before 2002, HepB was available in China but was not included in the National Immunization Program (NIP); families had to pay out-of-pocket for the vaccine. In 2002, China included HepB into its Expanded Program on Immunization (EPI), making the vaccine available at no cost to families. HepB was administered at 0, 1, and 6 months of age using a 5 µg recombinant yeast-derived vaccine (brand name Hepatitis B Vaccine Made by Recombinant DNA Technique in Yeast; adjuvant: aluminum). In 2012, China suspended production of the 5 µg vaccine, replacing it with a 10 µg vaccine in 2014 after supplies of the 5 µg vaccine were exhausted. Thus, a 5 µg HepB vaccine was administered to infants born from 2002 to 2013.

The World Health Organization (WHO) and the U.S. Centers for Disease Control and Prevention (CDC)^4^ do not recommend HepB booster vaccination in successfully immunized individuals. Some studies^5^ indicate that a full primary series of HepB confers protection against hepatitis B infection for long periods of time, even though anti-HBs antibody levels wane over time and eventually become undetectable in some successfully immunized individuals. However, it is controversial whether a decline in serum anti-HBs antibody levels implies reduced protection and the need for a booster dose^6,7^. China has a large number of persons living with HBV who therefore have potential to cause horizontal transmission of HBV. Horizontal transmission could be seen as an increase in acute hepatitis B (AHB) and HBsAg prevalence following vaccination during infancy. Evidence of horizontal transmission could indicate a need for a booster dose of HepB.

In China there was a voluntary practice of screening children for HBsAg and anti-HBs antibody levels at kindergarten and primary school entry and offering a booster dose to those negative for HBsAg and anti-HBs antibody. Using data from a 2014 national serological survey, we analyzed seropositivity rates of these two markers in 1–12-year-old children to assess for evidence of horizontal transmission and impact of the booster dose strategy on antibody levels. We evaluated the incidence of AHB by birth cohort to look for evidence of increased AHB in China. We report results of our analyses to provide evidence regarding the need for a booster dose for children vaccinated as infants with the 5 µg HepB vaccine.

## Material and methods

### Study design

The setting was mainland China. We assessed HBsAg prevalence by birth cohort using data from a national serological survey, and we assessed AHB incidence in the same birth cohorts to determine whether AHB or HBsAg prevalence were increasing after infancy. We compared anti-HBs antibody levels of children given a booster dose of HepB with those not given a booster dose to assess the impact of the booster dose on antibody levels.

### Data sources and participants

Children who participated in a 2014 HBV serological survey, described elsewhere^8^, were included in this study who met the following criteria: (1) born in 2002 to 2013, (2) vaccinated with a 5 µg HepB vaccine as a birth dose and primary series; and (3) having all three doses administered by 12 months of age.

In China, many children were voluntarily tested to determine anti-HBs antibody levels before entering kindergarten or primary school. Children negative for anti-HBs antibody would be offered a booster dose of HepB vaccine. It was up to the parents to decide whether or not to accept the offer. Among the 6% of subjects in the 2014 serological survey who had received booster doses, 85.57% received a booster dose between 2 and 6 years of age, and 11.08% received a booster dose after 6 years of age.

Coverage levels of HepB1-OT (defined as the percentage of newborn infants who received a dose of HepB within 24 h of birth) and HepB3 (defined as the percentage of children who received 3 doses of HepB before reaching 12 months of age) by birth cohort were obtained from the Children Immunization Information System (CIIMS) managed by the Chinese Center for Disease Control and Prevention (China CDC).

We determined the incidence of AHB among children over 5 years of age in the 2002–2007 birth cohorts using data from the National Notifiable Disease Reporting System (NNDRS) reported from 2008 to 2014. For example, children in the 2002 birth cohort were 6 to 12 years old in 2008–2014, the years for which their AHB incidence was obtained from NNDRS.

### Statistical analysis

Chi-square testing was used to compare HBsAg and anti-HBs seropositivity trends, and to compare seropositivity rates between booster and no-booster groups. P < 0.05 was considered statistically significant. We determined coverage of HepB1-OT and HepB3 from 2002 to 2013. AHB incidence was determined for each birth cohort.

### Ethical reviews

The 2014 HBV serosurvey was approved by China CDC’s Ethical Review Committee. Ethical approval was not required for analysis of routinely obtained coverage and disease incidence data, as these data were obtained in aggregate form without personal identifying information.

## Results

### HBsAg and anti-HBs seropositivity

In total, 12,713 children from the 2014 serological survey were included in this study, and among these children, 7,663 (63.1%) were born to HBsAg-negative mothers, 313 (2.6%) were born to HBsAg-positive mothers, and 4167 (34.3%) were children born to mothers with unknown HBsAg status. Among all children, 11,401 (93.9%) had not received a booster dose, and 742 (6.1%) had. Among children receiving a booster dose, 480 (64.7%) received one booster dose and the rest received 2 or more doses. The practice of screening and administering booster doses decreased over time (Table 1).

**Table 1:** Characteristics of the study population.

Characteristic	No booster		Booster		Total	
	N	%	N	%	N	%
**Birth year**						
2012–2013	3567	97.43	94	2.57	3661	100.00
2010–2011	4195	96.50	152	3.50	4347	100.00
2008–2009	1429	92.13	122	7.87	1551	100.00
2006–2007	1126	87.97	154	12.03	1280	100.00
2004–2005	447	85.14	78	14.86	525	100.00
2002–2003	637	81.77	142	18.23	779	100.00
**HBsAg of mother**						
Negative	7151	93.3	512	6.7	7663	100.00
Positive	279	89.1	34	10.9	313	100.00
Unknown	3971	95.3	196	4.7	4167	100.00
Total	11,401	93.9	742	6.1	12,143	100.00

The HBsAg-positivity rate among children who did not receive the booster was 0.41%, and It did not increase with age (χ^2^ trend = 0.698, p = 0.403). The HBsAg-positive rate among children born to HBsAg-positive mothers, HBsAg-negative mothers, and HBsAg-unknown mothers was 2.15%, 0.31%, and 0.48%, respectively. Children’s HBsAg positivity did not increase with age (χ^2^ Trend = 0.012, p = 0.911; χ^2^ Trend = 1.744, p = 0.187; χ^2^ Trend = 0.395, p = 0.53) (Table 2).

**Table 2:** HBsAg seropositivity among children without booster doses.

Birth year	HBsAg( +) mother			HBsAg(–) mother			HBsAg(unknown) mother			Total		
	N	+	%	N	+	%	N	+	%	N	+	%
2012–2013	102	1	0.98	2265	7	0.31	1200	5	0.42	3567	13	0.36
2010–2011	100	2	2.00	2697	9	0.33	1398	6	0.43	4195	17	0.41
2008–2009	33	0	0.00	829	2	0.24	567	2	0.35	1429	4	0.28
2006–2007	21	2	9.52	692	2	0.29	413	4	0.97	1126	8	0.71
2004–2005	10	1	10.00	270	0	0.00	167	1	0.60	447	2	0.45
2002–2003	13	0	0.00	398	2	0.50	226	1	0.44	637	3	0.47
Total	279	6	2.15	7151	22	0.31	3971	19	0.48	11,401	47	0.41

The anti-HBs positivity rate among children who did not receive the booster was 60.85%, and the rate declined with age (χ^2^ Trend = 555.37, *p* < 0.001). The anti-HBs positivity rate among children born to HBsAg-positive mothers, HBsAg-negative mothers, and HBsAg-unknown mothers was 69.18%, 61.71%, and 58.70% and also declined with age (χ^2^ Trend = 6.025, *p* = 0.014; χ^2^ Trend = 348.02, *p* < 0.001; χ^2^ Trend = 197.91, *p* < 0.001 (Table 3).

**Table 3:** Anti-HBs seropositivity among children without booster doses.

Birth year		HBsAg( +) mother			HBsAg(–) mother			HBsAg(unknown) mother			Total		
		N	+	%	N	+	%	N	+	%	N	+	%
2012–2013		102	80	78.43	2265	1729	76.34	1200	874	72.83	3567	2683	75.22
2010–2011		100	70	70.00	2697	1661	61.59	1398	856	61.23	4195	2587	61.67
2008–2009		33	17	51.52	829	413	49.82	567	266	46.91	1429	696	48.71
2006–2007		21	11	52.38	692	311	44.94	413	168	40.68	1126	490	43.52
2004–2005		10	6	60.00	270	120	44.44	167	73	43.71	447	199	44.52
2002–2003		13	9	69.23	398	179	44.97	226	94	41.59	637	282	44.27
Total		279	193	69.18	7151	4413	61.71	3971	2331	58.70	11,401	6937	60.85

The anti-HBs positivity rate among children who received boosters was 88.41% and declined with age (χ^2^ Trend = 14.62, *p* < 0.001). The anti-HBs positivity rate among children born to HBsAg-positive mothers, HBsAg-negative mothers, and HBsAg-unknown mothers was 91.18%, 89.26%, and 85.71%, respectively (Table 4).

**Table 4:** Anti-HBs seropositivity among children with booster doses.

Birth year		HBsAg(+) mother			HBsAg(–) mother			HBsAg(unknown) mother			Total		
		N	+	%	N	+	%	N	+	%	N	+	%
2012–2013		5	5	100	69	62	89.86	20	20	100.00	94	87	92.55
2010–2011		10	10	100	110	98	89.09	32	28	87.50	152	136	89.47
2008–2009		5	4	80	83	80	96.39	34	29	85.29	122	113	92.62
2006–2007		9	8	88.89	111	101	90.99	34	34	100.00	154	143	92.86
2004–2005		0	0	–	56	52	92.86	22	17	77.27	78	69	88.46
2002–2003		5	4	80	83	64	77.11	54	40	74.07	142	108	76.06
Total		34	31	91.18	512	457	89.26	196	168	85.71	742	656	88.41

None of the 742 children who received booster doses were positive to HBsAg. Among the 11,401 children who did not receive booster doses, the HBsAg positivity rate was 0.4%. There was no statistically significant difference in HBsAg positivity between the booster group and the no-booster group (χ^2^ = 3.1, *P* = 0.08). Regardless of the mother’s HBsAg status, there were no statistically significant differences in HBsAg positivity between the booster group and the no-booster group. Anti-HBs positivity in the booster-dose group was higher than in the no-booster group (Table 5).

**Table 5:** Comparison of the HBsAg positive rate and anti-HBs positive rate between the booster group and the no booster group.

Characteristics		HBsAg			Anti-HBs		
		+	–	*P*	+	–	*P*
HBsAg( +) mother	Booster	0 (0)	34 (100)	*P* = 0.5(Fisher)	31 (91.2)	3 (8.8)	*P* = 0.007
	No booster	6 (2.2)	273 (97.8)		193 (69.2)	86 (30.8)	
HBsAg(–) mother	Booster	0 (0)	512 (100)	*P* = 0.21	457 (89.3)	55 (10.7)	*P* < 0.001
	No booster	22 (0.3)	7129 (99.7)		4413 (61.7)	2738 (38.3)	
HBsAg(unknown) mother	Booster	0 (0)	196 (100)	*P* = 0.33	168 (85.7)	28 (14.3)	*P* < 0.001
	No booster	19 (0.5)	3952 (99.5)		2331 (58.7)	1640 (41.3)	
Total	Booster	0 (0)	742 (100)	*P* = 0.08	656 (88.4)	86 (11.6)	*P* < 0.001
	No booster	47 (0.4)	11,354 (99.6)		6937 (60.8)	4464 (39.2)	

### HepB1-OT and HepB3 coverage

**Figure 1:**
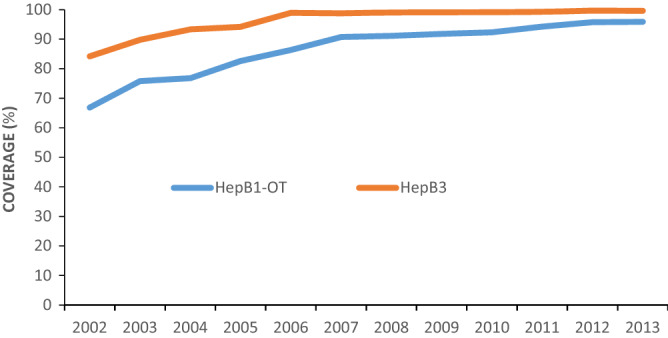
Annual HepB1-OT and HepB3 coverage in China, 2002–2013.

From 2002 through 2013, 514,893,249 doses of 5 µg-HepB were administered to children in China. The mean HepB1-OT coverage was 86.7% (Range: 66.8–95.9%), and the mean HepB3 coverage was 96.2% (Range: 84.2–99.7%). Coverage increased year by year (Fig. 1).

### AHB incidence, ages six to twelve

**Figure 2:**
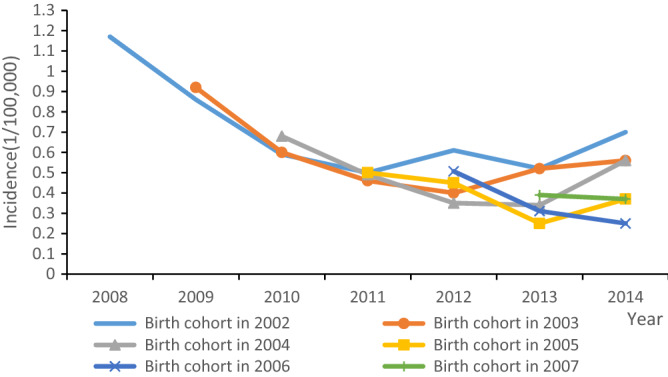
AHB incidence in the 2002 to 2007 birth cohorts.

Figure 2 shows the annual incidence of AHB by birth cohort during the years that the children birth cohorts were from 6 years old to up to 12 years of age. For example, one can see in the figure that the incidence of AHB did not increase with age among children born in 2002 from when they were 6 years old to when they were 12 years old (observation years 2008–2014; χ^2^ Trend = 0.273, p = 0.602). The χ^2^ trend test for the 2003 through 2006 birth cohorts also showed that there was no increasing trend of AHB (2003 χ^2^ trend = 0.238, p = 0.626; 2004 χ^2^ Trend = 0.164, p = 0.686; 2005 χ^2^ Trend = 0.322, p = 0.570; 2006 χ^2^ Trend = 0.79, p = 0.370).

## Discussion

Our study showed that based on the 2014 nationwide hepatitis B serological survey, HBsAg seropositivity did not increase with age among children who received the hepatitis B vaccine birth dose and two more HepB doses during infancy. Additionally, national surveillance for AHB showed that the incidence of AHB did not increase among children between 6 and up to 12 years of age. We believe that this provides supportive evidence that protection from a 5 µg antigen HepB is maintained through childhood, indicating that booster doses of HepB are not indicated.

Our study also showed that the practice of screening for HBsAg and anti-HBs antibodies at kindergarten and primary school entry and offering HepB booster doses to children negative for both (e.g., uninfected children with no detectable anti-HBs) was associated with increases in anti-HBs seropositivity. That these children remained uninfected may show protection, but also may be related to immunity despite lack of antibodies or to a lack of a horizontal force of infection due to very little circulation of HBV due to the low endemicity of HBV among children (0.41%). We believe that our findings do not provide evidence in support of the screening and boosting strategy.

That anti-HBs concentrations declined year by year in boosted and non-boosted children was observed in other study^9^. Although anti-HBs positivity decreased with age, protection against HBV is associated with immune memory, which persists beyond the time when anti-HBs disappears^10–12^.

Whether a booster dose of HepB is necessary for children born to HBsAg-positive mothers remains controversial. Some research indicated that individuals who were born to HBsAg-positive mothers had significantly increased risk of developing chronic HBV infection in adulthood, in which case booster doses may be appropriate for children born to HBsAg-positive mothers^13^. However, a 30-year prospective observational study performed in Hong Kong that initially enrolled 1,086 infants born to HBsAg-positive mothers and vaccinated after birth, showed that none developed chronic infection after 3 years of age^14^. In our study, although the HBsAg positive rate (2.15%) was significantly higher among children born to HBsAg-positive mothers than among children born to HBsAg-negative or HBsAg-unknown mothers within 1–12 years after primary vaccination, the HBsAg positivity rate did not increase with age. Our findings, therefore, do not provide evidence supporting use of a booster dose of HepB for children born to HBsAg-positive mothers.

China’s AHB surveillance data showed that the incidence of AHB in children in the birth cohorts of 2002 to 2007 also did not increase with age. We believe that this was due to implementation of the national vaccination program for newborns using a 5 µg HepB vaccine. The Hep3 coverage rate was greater than 80% since 2002. Apparently, the persistence of immunity from the 5 µg HepB was protective.

Six percent of children in the study received booster doses. China’s Ministry of Health does not recommend booster vaccination of children who received 3-doses of the 5 µg recombinant yeast-grown vaccine. Among the children who received booster doses, the HBsAg positivity rate was zero. This was likely because children were voluntarily tested for hepatitis B markers before entering kindergarten and primary school, and only if HBsAg and anti-HBs were both negative, would a booster dose be given. HBsAg positive children would not receive a booster dose. As one would anticipate, the anti-HBs positive rate was significantly higher in the booster group than in the no-booster group, a finding shown by others^15,16^. There was no statistically significant difference in HBsAg positivity between the two groups. A study from Taiwan showed that booster vaccination against hepatitis B did not confer additional protection against becoming an HBsAg carrier^17^.

Strengths of the study are that the data were nationally-representative with large sample sizes, including for the 2014 sero-epidemiological survey. Limitations are that the longest follow-up available is 12 years and that the HBsAg status of mothers in the serological survey were from self-report, leading to the high proportion of unknown HBsAg status mothers.

This study showed that in spite of subsequent decline of detectable serum anti-HBs, a full primary course of 5 µg HepB conferred apparently complete protection against acute clinical disease—evidence supportive of not using a screening/booster dose strategy for all infants. This is different than for infants born to HBsAg positive women. The HBsAg positivity rate among women of childbearing age is still relatively high^18,19^, and infants born to HBsAg-positive mothers who are HBsAg negative with anti-HBs levels < 10 mIU/mL after having received a complete, 3-dose HepB vaccine series should be identified by post-vaccination serological 1–2 months after the third dose of HepB vaccine. Infants born to HBsAg positive women who did not respond to HepB should be revaccinated with a second 3-dose HepB vaccine series. Periodic serological surveys and AHB surveillance among children in the 2002 to 2012 birth cohorts should continue to be strengthened.

## Supplementary information

Supplementary Information.
