# Supplementary material for: Protective effect of vaccinating infants with a 5 µg recombinant yeast-derived hepatitis B vaccine and the need for a booster dose in China
Source: Sci Rep. 2020 Oct 23;10:18155. doi: 10.1038/s41598-020-75338-5 (PMC7584599; doi:10.1038/s41598-020-75338-5)
Supplement: Supplementary file 1 — Supplementary Information. [file 41598_2020_75338_MOESM1_ESM.pdf]

Protective effect of vaccinating infants with a 5μg recombinant yeast-derived hepatitis B and the need for a booster dose in China

Ning Miao[1], Hui Zheng[1], Xiaojin Sun[1], Guomin Zhang[1], Fuzhen Wang[1*]

[1]Chinese Center for Disease Control and Prevention, Nanwei Road, Xicheng District, Beijing
*email: wangfz@chinacdc.cn

Supplementary information

a:China conducted a HBV serosurvey in 2014.Target populations for 2014 survey were local residents residing in national disease surveillance points (DSPs) for>6 months. DSPs were selected by China CDC to be representative of the population of China. The final sample was 31,713 persons. Among them, 19,900 were born in the birth cohort from 2002 to 2013, accounting for 62.8%.

In the 2014 serosurveys ,face-to-face interviews with the respondent or the respondent's parent were completed by trained staff by using standard questionnaires to obtain basic information,including birthdate, HepB vaccination history (validated by parent-held certificate or village vaccination record) and HBV status of mother(self-report).

All specimens in the 2014 serosurveys were tested in the National Hepatitis Laboratory of the Institute for Viral Disease Control and Prevention, China CDC . ELISA reagents were used to detect levels of HBsAg and anti-HBs. Specimens yielding inconsistent or indeterminate results were retested by using microparticle enzyme immunoassay reagents (Abbott Laboratories, Chicago, IL, USA).
